# Targeting metabolic vulnerabilities in breast cancer cells by combining PEDF and doxorubicin: pathway insights from GC/MS-based metabolomics

**DOI:** 10.17179/excli2025-8508

**Published:** 2025-08-18

**Authors:** Raziyeh Abooshahab, Hani Al-Salami, Crispin R. Dass

**Affiliations:** 1 Curtin Medical School, Curtin University, Bentley 6102, Australia; 2 Curtin Health Innovation Research Institute, Bentley 6102, Australia; 3 Biotechnology and Drug Development Research Laboratory, Curtin Health Innovation Research Institute, Bentley 6102, Australia; 4 Faculty of Pharmacy, Silpakorn University, Nakhon Pathom 73000, Thailand

**Keywords:** breast cancer, PEDF, doxorubicin, GC/MS, metabolomics, metabolic profiling

## Abstract

Breast cancer (BC), characterised by its diverse subtypes and molecular heterogeneity, remains a major challenge in oncology. Despite advances in chemotherapy, such as doxorubicin (Dox), limitations persist due to toxicity and drug resistance. Pigment epithelium-derived factor (PEDF) is a multifunctional protein with unique anti-tumour properties. The aim here was to elucidate metabolic reprogramming in human BC cell lines using a metabolomics approach. Untargeted gas chromatography-quadrupole mass spectrometry (GC/Q-MS) was employed to identify the metabolic alterations in BC cell lines MCF-7 (ER-positive) and MDA-MB-231 (TNBC) following treatment with PEDF, Dox, and their combination (Dox+PEDF) in comparison to untreated controls. Statistical models were employed using a combination of multivariate and univariate analyses, including partial least squares discriminant analysis (PLS-DA) and one-way ANOVA, applied by MetaboAnalyst and SIMCA software. To address the potential for multiple-testing errors, false discovery rate (FDR)-adjusted p-values were calculated to ensure robust statistical reliability. The overall analysis revealed significant metabolic alterations across the treatment groups, with distinct patterns emerging in carbohydrate, lipid, and amino acid metabolisms. In MCF-7 cells, PEDF combined with Dox significantly decreased cystine levels and modulated aspartic acid and lipid-related metabolites, indicating potential shifts in redox homeostasis and membrane composition. In MDA-MB-231 cells, the combination treatment significantly reduced glucose-6-phosphate and lactate levels, suggesting remodeling of glycolytic flux and redox balance. Furthermore, the combination of PEDF and Dox influenced amino acid and lipid metabolism. Pathway enrichment and correlation analyses revealed significant perturbations in glutathione metabolism, energy pathways, and lipid signaling, with notable differences between the two cell lines. Combining Dox and PEDF induced coordinated changes in metabolic networks, suggesting synergistic and antagonistic mechanisms that impact multiple biochemical pathways. These findings underline the importance of combining PEDF with chemotherapy to improve treatment outcomes in BC.

See also the graphical abstract(Fig. 1).

## 1. Introduction

Breast cancer (BC) remains a critical challenge in oncology due to its biological complexity and diverse subtypes (Zubair et al., 2021[47]). The classification of BC is largely based on the presence or absence of specific receptors, including those for estrogen (ER), progesterone (PR), and human epidermal growth factor (HER2). Subtypes like luminal (ER/PR positive), HER2-positive, and triple-negative breast cancer (TNBC; which lacks all three receptors) exhibit distinct clinical behaviours and treatment responses (Dent et al., 2007[17], Cancer Genome Atlas Network, 2012[13]). Despite recent advancements in targeted therapies, effective treatment of BC remains challenging due to its propensity for treatment resistance and the risk of recurrence (Abooshahab et al., 2024[1]). This highlights the urgent need for continuous innovation in treatment strategies to combat this disease.

Current treatment strategies often rely on chemotherapy, such as doxorubicin (Dox), a well-known anthracycline valued for its potent anti-tumour activity through DNA intercalation and inhibition of topoisomerase II, ultimately leading to apoptosis in unbridled cells (Tacar et al., 2013[41]). However, resistance to chemotherapy and the adverse side effects associated with Dox pose substantial obstacles in clinical practice. Consequently, exploring combination therapies that can enhance Dox efficacy while minimising toxicity has become a focal point in cancer research. Pigment epithelium-derived factor (PEDF), a multifunctional protein with anti-angiogenic, anti-inflammatory, and anti-tumour properties (Abooshahab et al., 2021[2]), has shown promise in enhancing the anti-cancer effects of Dox while potentially reducing its toxicity. Our group has previously found that PEDF may help alleviate some of Dox's side effects on vital organs, including the heart, small intestine, and testes, indicating a protective effect that could support combination therapy (Tacar et al., 2015[40]). In addition, our recent study further explored the combined effects of Dox and PEDF on human BC cells, revealing that the Dox+PEDF treatment significantly impacts signaling pathways and metastatic markers expression across different BC types (Abooshahab et al., 2024[1]). However, the precise biochemical mechanisms underlying the combined action of Dox and PEDF remain largely unexplored.

Building on those findings, this study employed metabolomics profiling to characterise the effects of Dox and PEDF combination on BC cells. Metabolomics, the comprehensive analysis of small molecules, provides a unique window into the biochemical landscape of cancer cells, revealing insights into the metabolic adaptations that drive cancer progression and treatment response (Beger, 2013[10], Abooshahab et al., 2024[5]). Unlike other omics approaches, metabolomics provides direct insights into cellular function and biochemical activity, capturing real-time physiological responses to treatment (Beger, 2013[10], Schmidt et al., 2021[35]). Metabolomics studies on Dox-treated cancer cells have revealed significant alterations in pathways related to amino acid metabolism, energy production, and oxidative stress (Alsherbiny et al., 2021[7], Alarcon-Zapata et al., 2023[6], Govindasamy et al., 2023[22], Liu et al., 2023[28], Rushing et al., 2023[34]). More recently, studies by our team that utilised metabolomics to investigate PEDF's effects on BC cells have reported alterations in pathways associated with glucose metabolism, lipid processing, and amino acid synthesis (Abooshahab et al., 2023[3], Abooshahab et al., 2023[4]). 

In this study, we employed gas chromatography-mass spectrometry (GC/MS)-based metabolomics to investigate the metabolic alterations in two human BC cell lines, MCF-7, an ER-positive luminal subtype, and MDA-MB-231, a TNBC subtype, following treatment with PEDF, Dox, and their combination. These cell lines are widely used in vitro studies and offer robust models for exploring key aspects of BC biology due to their contrasting phenotypic and genotypic profiles. By elucidating the unique and shared metabolic pathways affected by these treatments, we aimed to find the molecular mechanisms driving the potential synergy or antagonism between PEDF and Dox at biochemical levels. Such insights could pave the way for more effective and less toxic treatment strategies for BC, ultimately improving clinical outcomes for patients with different BC subtypes.

## 2. Materials and Methods

### 2.1. Materials

Recombinant PEDF was purchased from MD Bioproducts (Bethesda, MD, USA). The MCF-7 and MDA-MB-231 cell lines were obtained from the American Tissue Culture Collection, ATCC (Manassas, VA, USA). Dulbecco's modified Eagle medium (DMEM), foetal bovine serum (FBS), and antibiotic/antimycotic were all purchased from Sigma-Aldrich, Saint Louis, MO, USA. HPLC-grade Doxorubicin (Dox), isopropanol (IPA), methanol (MeOH), 4,4′-dibromooctafluorobiphenyl, and water (H_2_O) were obtained from Sigma-Aldrich (Saint Louis, MO, USA). Derivatisation reagents, including methoxyamine (MOX), trifluoroacetamide (MSTFA), and 1% trimethylsilyl chloride (TMCS), were purchased from Thermo Fisher Scientific, Waltham, MA, USA.

### 2.2. Cell Cultivation and Harvesting for Metabolomics Analysis

The human luminal-A (ER+/PR+/−/HER2−) breast adenocarcinoma cell line (MCF-7) and the human triple-negative breast cancer cell line (MDA-MB-231) were cultured in DMEM with 10% foetal bovine serum (FBS) and 1% antibiotics and antimycotics under normal glucose conditions (5 mM). The cultures were grown to 80% confluence under controlled conditions at 37 °C with 5% CO2. 

For metabolomics studies, MCF-7 and MDA-MB-231 cells were seeded at densities of 4 × 10⁵ cells/well and 35 × 10⁵ cells/well, respectively, in 24-well plates. After 24 h of culture, cells were exposed to one of the following treatments: PEDF (100 nM) (Filiz et al., 2012[19]), Dox (0.5 µM) (Aniogo et al., 2017[9]), or a combination of PEDF and Dox, with untreated cells as the control group, and incubated overnight. After 24 h, the cells underwent trypsinisation and were centrifuged at 700 g for 5 min. The culture medium was discarded, and the cell pellets were rapidly frozen in liquid nitrogen to quench metabolism and stored at −80 °C until sample preparation. The experiment was carried out with five replicates per treatment group and two technical replicates.

### 2.3. Sample Processing for Metabolomics Profiling

Metabolite extraction and derivatisation were performed following a previously described method with a few modifications (Abooshahab et al., 2023[3], Abooshahab et al., 2023[4]). Briefly, 500 µL of a chilled protein precipitation solution (a mixture of methanol, isopropanol, and water) 2:2:1 (v/v/v) was added to each sample. Then the samples were thoroughly mixed and kept at 4 °C for 20 minutes to facilitate precipitation. Subsequently, the mixtures were centrifuged at 21,952 g for 15 minutes at 4 °C. The resulting supernatants, containing cellular metabolites, were collected and dried under a nitrogen stream using a 24-position MICROVAP evaporator. The dried samples underwent a two-step derivatisation process. First, methoxyamine hydrochloride (40 μL) was added, and the mixture was incubated at 60 °C for 1 hour to achieve methoxyamination. In the second step, MSTFA, containing 1% TMCS as a trimethylsilylation agent, was introduced, and the reaction was completed by incubating the mixture at 45 °C for 20 minutes. Subsequently, each sample was dissolved in 20 µL of an injection standard consisting of 4,4′-dibromooctafluorobiphenyl at a concentration of 10 mg/L in hexane. The supernatant from each sample was promptly transferred into glass vials compatible with the autosampler for GC/MS analysis.

### 2.4. Metabolomics Data Acquisitions Using GC/MS 

An Agilent 5977B MSD/Agilent 8860 GC system fitted with a Restek Rxi-5-ms column (30 m length × 0.25 mm internal diameter (id); 0.25 μm film) was used to analyse the derivatised samples. Approximately 1 µL of each sample was injected at a split ratio of 1:1 in random order. The chromatographic method was conducted at a constant flow rate of helium, 1 mL/min, as the carrier gas. The initial oven temperature was held at 50 °C for 1 min, ramping from 20 °C/min to 320 °C, then held at 320 °C for 5 min. The MS source, transfer line, and quadrupole temperature were set at 150 °C, 290 °C, and 250 °C, respectively, operating in electron ionisation mode at −70 eV. After a 5.2 min solvent delay, mass spectrometry data were collected at a scan rate of 20 spectra/sec within the m/z 50-600 range.

### 2.5. Data Processing and Statistical Analysis

The raw data files were converted to ABF format using a free conversion tool (Lai et al., 2018[27]). The data were processed with MS-DIAL (version 4.9) to generate a comprehensive data matrix, including InChIKey identifiers, peak intensities, and the average retention time (RT) from the original dataset. The parameters used in MS-DIAL are provided in Table S1. Metabolite names were assigned to the GC/MS spectra based on mass spectral similarity and verified against multiple spectral libraries, such as the Fiehn library, MassBank with a ≥70% similarity threshold, and the Human Metabolome Database (HMDB) for precise metabolite identification. To ensure data accuracy and quality, contaminant ions derived from derivatisation reagents were excluded from the dataset. Peak intensities of duplicate features were summed to avoid redundancy. Blank reduction was performed to eliminate noise and ensure robust metabolite detection.

To normalise peak intensities, sum normalisation and autoscaling were applied on both cell lines using MetaboAnalyst (v6.0), ensuring a near-normal distribution of values. Multivariate statistical analyses, including partial least squares-discriminant analysis (PLS-DA), were performed in SIMCA-P 14.0 (Umetrics, Umeå, Sweden) to distinguish metabolic variations between experimental groups. Metabolites with variable importance in projection (VIP) minimum scores above 0.8 were deemed significant for group discrimination (Cox et al., 2013[16]). Model reliability was assessed through cross-validated predictive residuals, CV-ANOVA, and permutation tests. To identify metabolites significantly influenced by experimental factors, one-way ANOVA was conducted on the annotated GC/MS dataset, with p-values adjusted for multiple testing using false discovery rate (FDR) correction. Metabolite intensities were visualised as box plots using the R packages “ggpubr” and “tidyverse.” Additionally, Pearson correlation coefficients were computed to explore pairwise correlations among all metabolites, displayed as a heatmap via the “corrplot” R package. Finally, primary biological pathways were examined through enrichment pathway analysis in MetaboAnalyst (v6.0), with significance thresholds set at p-value < 0.05. 

## 3. Results

### 3.1. Metabolic Profiles Among the Groups

**Figure 2:**
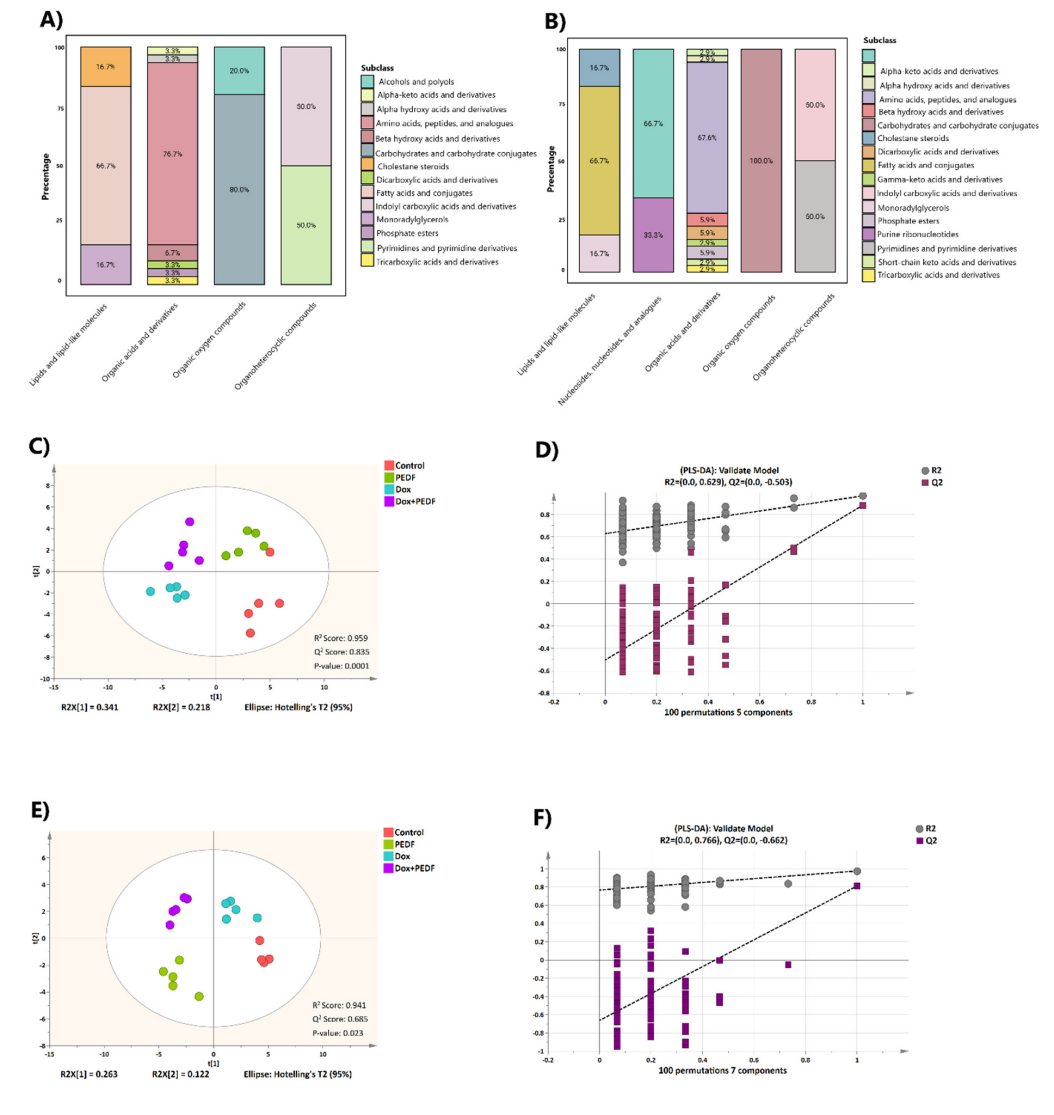
Figure 2. Metabolic features of breast cancer cell lines. A, B) Stacked bar charts representing the distribution of metabolite subclasses by percentage across different metabolite categories. The PLS-DA score plots visualise group separation among control, PEDF, Dox, and Dox+PEDF groups in MCF-7 (C) and MDA-MB-231 (E). Each point represents a sample, colour-coded by treatment group. The R² and Q² values indicate the model's explained variance and predictability, respectively, with significant group clustering (*p *< 0.05). D, F) Permutation tests as a validation plot for the models displaying R² and Q² values for 100 permutations. The values demonstrate the model's robustness and predictive accuracy.

The metabolomics data from the MCF-7 and MDA-MB-231 cell lines were processed separately using MS-DIAL, identifying a total of 368 compounds for MCF-7 and 380 compounds for MDA-MB-231. From these, 43 metabolites for MCF-7 and 50 metabolites for MDA-MB-231 were considered reliable for further analysis. The identified metabolites for each cell line were then categorised using the ClassyFire classification system (Djoumbou Feunang et al., 2016[18]). At the superclass level, the metabolic profiles of MCF-7 and MDA-MB-231 cells revealed a predominance of organic acids and derivatives, accounting for the majority of metabolites, 69.76% in MCF-7 and 68% in MDA-MB-231. This was followed by lipids and lipid-like molecules, comprising 13.95% in MCF-7 and 12% in MDA-MB-231. Organic oxygen compounds comprised 11.62% in MCF-7 and 10% in MDA-MB-231 (Figure 2A, 2B(Fig. 2)). At the subclass level, amino acids, peptides, and their analogs dominated both cell lines, representing 53.5% of the metabolites in MCF-7 and 47.9% in MDA-MB-231. Tables S2 and S3 provide a detailed list of the identified metabolites, including their respective classes. 

We employed supervised PLS-DA models to identify the metabolites that distinguished the groups in both cell lines. The PLS-DA score plots exhibited distinct and well-separated clusters for the four groups, with acceptable levels of explained variance for MCF-7 and MDA-MB-231, highlighting significant metabolic alterations across the samples (Figure 2C, 2E(Fig. 2)). The models were statistically significant, as evidenced by CV-ANOVA (p < 0.05). Additionally, the predictive performance of the models was validated through a permutation test (n = 100), where the original R² and Q² values exceeded those of the permuted models, confirming the models' reliability and predictive strength (Figure 2D, 2F(Fig. 2)).

### 3.2. Response of MCF-7 Metabolomes to PEDF, Dox, and Dox+PEDF

**Figure 3:**
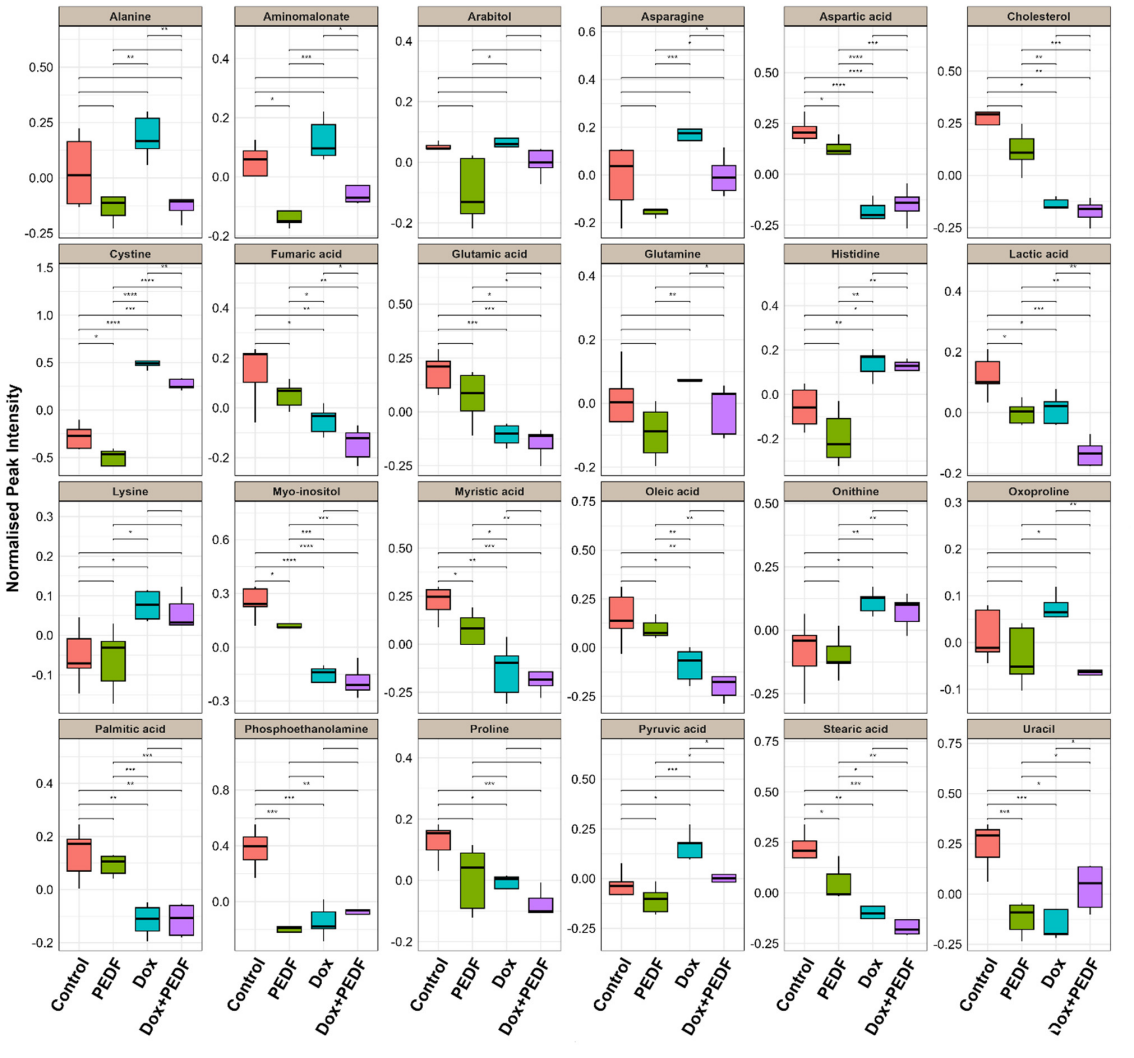
Figure 3. Box plots for significantly altered metabolites in MCF-7 cells. The boxplots display the distribution of 24 metabolites in MCF-7 cells that exhibited the highest significance (*p*-values < 0.05 and VIP scores ≥ 0.8) in the analysis of variance. These boxplots enable comparison of the four groups: Control, PEDF, Dox, and Dox+PEDF. On the *x*-axis, each group is represented by individual metabolites, while the *y*-axis indicates the normalised peak intensity. Metabolites showing significant differences were calculated using Tukey's Honestly Significant Difference (Tukey HSD) test and indicated as (*) *p* ≤ 0.05, (**) *p* ≤ 0.01, and (***) *p* ≤ 0.001.

A one-way ANOVA (p < 0.05, FDR q < 0.05) followed by multivariate analysis (VIP score > 0.8) identified 24 significantly altered metabolites in MCF-7 from a total of 43 metabolites (Table 1(Tab. 1)). These metabolites demonstrated marked differences across the four experimental groups, particularly impacting glycolysis, amino acid metabolism, and lipid biosynthesis. Box plots were generated for the most statistically significant metabolites to provide a clear visualisation of the group-specific variations in metabolite abundance, as shown in Figure 3(Fig. 3).

Cystine showed the most notable variation among the treatment groups (q = 1.23E-09, VIP = 1.100). Compared to the control group, a significant decrease and increase were observed in the PEDF and Dox groups. The combination of Dox and PEDF reduced cystine level compared to Dox treatment alone, although it remained elevated relative to the control. This suggests a potential antagonist interaction between Dox and PEDF in lowering cystine levels, reflecting alterations in redox homeostasis. Aspartic acid was significantly reduced in the PEDF, Dox, and Dox+PEDF groups (q = 1.46E-06, VIP = 0.986) compared to the control group. Interestingly, in the combination group (Dox+PEDF), a slight increase in aspartic acid levels was observed compared to the Dox treatment alone, suggesting a potential modulatory effect of the combined treatment on aspartic acid metabolism. Additionally, amino acids such as glutamic acid and proline showed significant reductions, particularly in the Dox+PEDF group, indicating that amino acid metabolism is extensively affected, possibly influencing cell growth and apoptosis. Myo-inositol levels decreased across all three treatment groups (PEDF, Dox, and Dox+PEDF) compared to the control (q = 2.30E-06, VIP = 0.985), with a further reduction observed in the combination group. Lipid-related metabolites, including phosphoethanolamine, palmitic acid, and myristic acid, were significantly altered and responded differently to the treatment groups. On the other hand, the treatments significantly impacted cholesterol levels (q = 0.0003, VIP = 1.005), particularly in the Dox and Dox+PEDF groups.

### 3.3. Response of MDA-MB-231 Metabolomes to PEDF, Dox, and Dox+PEDF 

The same analysis was performed for MDA-MB-231 to find substantially altered metabolites among groups. Of 50 metabolites, 23 were significant in MDA-MB-231 (Table 2(Tab. 2)). Among all significantly altered metabolites in MDA-MB-231 cells, glucose-6-phosphate (G6P) exhibited the most considerable variation across treatments. There was a marked reduction in G6P levels in the PEDF group compared to control, Dox, and Dox+PEDF treatments (q = 1.22E-09, VIP = 1.379). Dox exposure resulted in a slight increase in G6P levels, whereas the combination of Dox+PEDF led to a significantly greater reduction in G6P levels than Dox alone. Lactate levels were also significantly altered across the treatment groups (q = 8.42E-05, VIP = 1.378). PEDF treatment resulted in the lowest lactate levels, while Dox treatment significantly increased lactate levels compared to the control. Interestingly, the combination of Dox + PEDF caused a reduction in lactate levels compared to Dox alone; however, this reduction did not reach statistical significance. For inosine and ribulose-5-phosphate, the most pronounced increases were observed in the Dox+PEDF group. PEDF treatment alone also significantly elevated these metabolites compared to the control. Still, the combination treatment exerted a more robust effect compared to PEDF alone, suggesting a synergistic interaction between Dox and PEDF on nucleotide metabolism and oxidative stress pathways. Similar trends were observed for the amino acids glycine and lysine, which significantly increased following PEDF treatment compared to the control. However, Dox treatment reduced the levels of these amino acids. Importantly, the combination of Dox+PEDF led to significantly higher levels of glycine and lysine than Dox alone, suggesting that the combination therapy exerts an additive effect on amino acid metabolism. Glutamic acid levels were significantly elevated across the PEDF, Dox, and Dox+PEDF groups compared to the control (q = 0.0006, VIP = 1.018). The Dox+PEDF combination induced the highest glutamic acid levels, which were significantly greater than those observed with Dox alone. This highlights the synergistic effect of the combination treatment on glutamic acid accumulation. 

**Figure 4:**
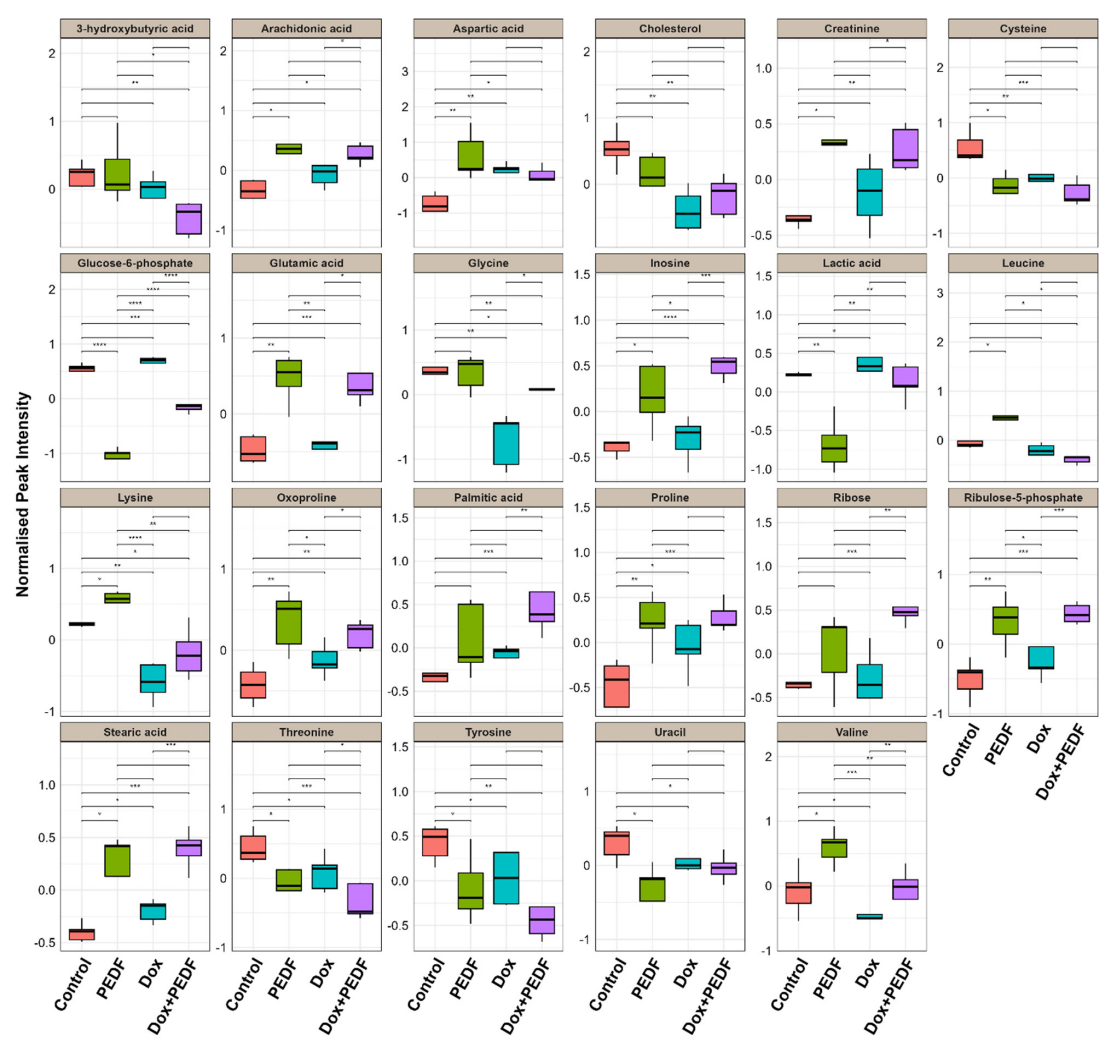
Box plots for significantly altered metabolites in MDA-MB-231. The boxplots display the distribution of 23 metabolites in MDA-MB-231 cells that exhibited the highest significance (*p*-values < 0.05 and VIP scores > 0.8) in the analysis of variance. These boxplots allow for comparing the four groups: Control, PEDF, Dox, and Dox+PEDF. On the *x*-axis, each group is represented by individual metabolites, while the *y*-axis indicates the normalised peak intensity. Metabolites showing significant differences were calculated using Tukey's Honestly Significant Difference (Tukey HSD) test and indicated as (*) *p* ≤ 0.05, (**) *p* ≤ 0.01, and (***) *p* ≤ 0.001

Both stearic acid (q = 0.0007, VIP = 1.021) and palmitic acid (q = 0.0140, VIP = 0.963) were significantly elevated in all treatment groups compared to control, with the Dox+PEDF combination showing the most evident effect. The levels of these fatty acids were significantly higher in the combination group compared to either Dox or PEDF alone. On the other hand, cholesterol levels were significantly reduced in the Dox group compared to the control. However, the Dox+PEDF combination partially restored cholesterol levels, although they remained below those of the control group. This partial recovery significantly differed from the Dox group alone, suggesting that PEDF may mitigate Dox-induced lipid dysregulation. Box plots were created for the most statistically significant metabolites to illustrate the group-specific variations in metabolite abundance, as depicted in Figure 4(Fig. 4).

### 3.4. Significant Metabolite Correlations in MCF-7 and MDA-MB-231 Cells Following Treatments

**Figure 5:**
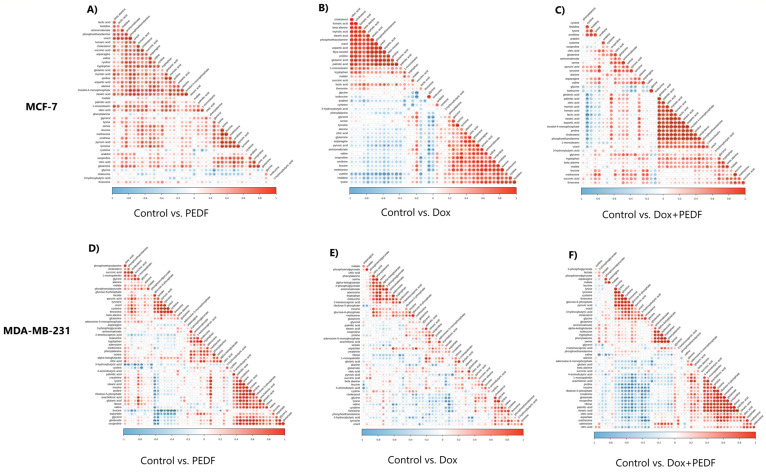
Figure 5. Metabolite-metabolite correlations in MCF-7 and MDA-MB-231 cells. A, B, C) Correlation matrices for MCF-7 cells and D, E, F) correlation matrices for MDA-MB-231 cells comparing Control vs. PEDF, control vs. Dox, and control vs. Dox+PEDF, wherein non-significant correlations (*p* > 0.05) are left uncoloured. Significant interactions (*p < 0.05, **p < 0.01, ***p < 0.001) are colour-coded, with positive correlations in red and negative correlations in blue.

The correlation analysis of MCF-7 and MDA-MB-231 cells treated with PEDF, Dox, and the combination of Dox+PEDF revealed a complex metabolic interplay characterised by significant positive and negative correlations. In MCF-7 cells, 861 metabolic correlations were found among treatment comparisons, revealing distinct interaction patterns. For the control vs. PEDF group (Figure 5A(Fig. 5)), 389 positive and 10 negative correlations were identified. The control vs. Dox comparison (Figure 5B(Fig. 5)) showed 404 positive and 102 negative correlations, while the control vs. Dox+PEDF group (Figure 5C(Fig. 5)) exhibited 318 positive and 70 negative correlations.

 In control vs. PEDF analysis, strong positive correlations were observed, including cholesterol with aminomalonate (r = 0.743), cholesterol with myo-inositol (r = 0.703), and fumaric acid with myo-inositol (r = 0.853). Notable negative correlations included 1-monostearin with cysteine (r = −0.723) and glycerol with glycine (r = −0.817). For the control vs. Dox group, prominent positive correlations were identified, such as cholesterol with aminomalonate (r = 0.743), cholesterol with oleic acid (r = 0.759), and fumaric acid with succinic acid (r = 0.837). Significant negative correlations included isoleucine with glycerol (r = −0.667) and glycine with glycerol (r = −0.817). In the control vs. Dox+PEDF comparison, key positive correlations were found between cholesterol and beta-alanine (r = 0.671) and cholesterol with myo-inositol (r = 0.703). Strong negative correlations were observed between glycine and glycerol (r = −0.817) and between uracil and 1-monostearin (r = −0.689).

In MDA-MB-231 cells, 1,225 metabolic correlations were identified across treatment groups. The control vs. PEDF group revealed 359 positive and 84 negative correlations (Figure 5D(Fig. 5)). Noteworthy positive correlations included those between cholesterol and cysteine (r = 0.802), cholesterol and pyruvic acid (r = 0.846), and succinic acid and phosphoethanolamine (r = 0.817). Prominent negative correlations were observed between cholesterol and leucine (r = −0.844), 3-hydroxybutyric acid and oleic acid (r = −0.849), and cystine and glycine (r = −0.884). The control vs. Dox comparison showed 184 positive and 86 negative correlations (Figure 5E(Fig. 5)). Significant positive correlations included oxoproline with glutamate (r = 0.961), oxoproline with glycerol (r = 0.919), and 1-monopalmitin with arachidonic acid (r = 0.776). Strong negative correlations featured glucose-6-phosphate with proline (r = −0.793), glycine with glycerol (r = −0.750), and glutamic acid with lactic acid (r = −0.777). For the Control vs. Dox+PEDF group, 348 positive and 208 negative correlations were identified (Figure 5F(Fig. 5)). Key positive correlations included 1-monopalmitin with ribose (r = 0.756), cholesterol with glycine (r = 0.638), and oxoproline with proline (r = 0.884). Notable negative correlations were cystine with glycine (r = −0.884), 3-hydroxybutyric acid with 1-monopalmitin (r = −0.722), and succinic acid with leucine (r = −0.750).

### 3.5. Pathway Analysis for MCF-7 and MDA-MB-231 Cells Following Treatments

**Figure 6:**
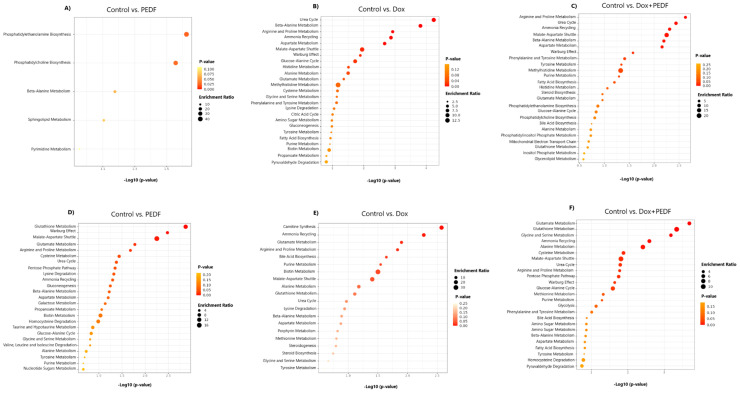
Figure 6. Metabolic Pathway Enrichment Analysis in MCF-7 and MDA-MB-231 Cells. The figure shows metabolic pathway enrichment analysis for MCF-7 (A, B, C) and MDA-MB-231 (C, E, F) cells under different treatments (Control vs. PEDF, Dox, and Dox+PEDF). Bubble size reflects the enrichment ratio, and bubble colour represents *p*-values, ranging from yellow (less significant) to red (more significant). The largest circles indicate the highest enrichment ratios, highlighting the key metabolic pathways that are significantly impacted by each treatment.

Pathway analyses of significant metabolites across treatment groups in each cell line were conducted using MetaboAnalyst (v.6.0). All enriched pathways can be found in Tables S4 to S9. The pathway analysis revealed significant metabolic alterations in MCF-7 and MDA-MB-231 cell lines across various treatment conditions. In the MCF-7 cell line, treatment with PEDF (Figure 6A(Fig. 6)) primarily influenced phosphatidylethanolamine and phosphatidylcholine biosynthesis pathways. In contrast, Dox treatment (Figure 6B(Fig. 6)) elicited marked enrichment in pathways related to amino acid metabolism, including the urea cycle, beta-alanine metabolism, and arginine and proline metabolism, emphasising significant metabolic perturbations. The combination of PEDF and Dox (Figure 6C(Fig. 6)) demonstrated an even broader range of enriched pathways, with notable involvement of arginine and proline metabolism, the urea cycle, and glutamate metabolism, suggesting a potential synergistic or additive effect that extends the metabolic impact observed with individual treatments. 

In the MDA-MB-231 cell line, PEDF treatment (Figure 6D(Fig. 6)) prominently enriched pathways such as glutathione metabolism, the Warburg effect, and the malate-aspartate shuttle, underscoring significant metabolic reprogramming in this more aggressive phenotype. Dox treatment (Figure 6E(Fig. 6)) showed pronounced enrichment in pathways, including carnitine metabolism, glutamate metabolism, ammonia recycling, arginine and proline metabolism, and bile acid biosynthesis. Notably, the combination of Dox and PEDF (Figure 5F(Fig. 5)) yielded the most extensive enrichment, particularly in glutamate metabolism, glutathione metabolism, and glycine and serine metabolism, reflecting the greater impact on these pathways under combination therapy.

## 4. Discussion

Breast cancer treatment is often constrained by the side effects associated with Dox, a widely used chemotherapeutic agent, which can compromise therapeutic efficacy (Belger et al., 2024[11]). To address this limitation, combination therapies are being explored to enhance treatment outcomes while mitigating adverse effects (Jiang et al., 2017[23], Abooshahab et al., 2024[1]). PEDF, a serine protease inhibitor, has demonstrated potential for inhibiting tumour growth and metastasis (Abooshahab et al., 2021[2]). Recent studies have indicated that PEDF can enhance the efficacy of Dox in BC cells by hindering tumour cell migration and impacting signaling pathways regarding tumour progression, suggesting a synergistic interaction between PEDF and Dox (Jones et al., 2023[24], Abooshahab et al., 2024[1]). However, the precise metabolic mechanisms underlying this modulation by PEDF remain inadequately understood. Given this, GC/MS-based metabolomics was employed to determine the combined effects of PEDF and Dox on BC cellular metabolic alterations. Our data revealed that alterations in metabolite levels distinctly exhibited unique response patterns in one cell line but not in the other. These differences underscore the metabolic divergence between the two BC cell lines and provide insights into potential metabolic vulnerabilities specific to each. Combining Dox and PEDF impacts energy production, amino acid metabolism, and antioxidant defences, suggesting a multifaceted approach to overcoming cancer cell metabolic resilience.

Glycolysis is one of the central metabolic pathways in cancer, where many aggressive tumours exhibit the "Warburg effect," relying on glycolysis for energy, even in the presence of oxygen (Vander Heiden et al., 2009[43], Potter et al., 2016[33]). This metabolic shift facilitates rapid ATP production and supplies intermediates for anabolic pathways crucial for cell growth and proliferation (Potter et al., 2016[33]). Lactate, the end product of glycolysis, is also generated to create an acidic tumour microenvironment, promoting invasion, immune evasion, and metastasis (Sharma et al., 2022[36]). Our study showed that Dox treatment led to opposing effects on lactate levels in the two cell lines, directly reflecting differences in glycolytic activity. In MDA-MB-231 cells, Dox increased lactate production, indicating an upregulation of glycolysis. This is a common adaptive response in aggressive cancer cells, where glycolysis provides not only energy but also a continuous supply of NAD+ through lactate dehydrogenase, necessary for maintaining glycolytic flux. This result reflects those of Maria et al. (2017[29]), who found that Dox increased the single pulse of lactic acid using High-Resolution Magic Angle Spinning (^1^H HR-MAS) NMR (Maria et al., 2017[29]). In contrast, MCF-7 cells exhibited a decrease in lactate production upon Dox treatment, indicating a shift away from glycolysis. This finding is consistent with *Karim et al*., who showed that Dox reduced intracellular lactate levels (Karim et al., 2024[25]). MCF-7 cells are generally less glycolytically active and more oxidative in nature, which may render them less capable of switching to glycolysis in response to Dox. When PEDF is combined with Dox, both cell lines exhibit reduced lactate levels. This also accords with our earlier observations, which showed that PEDF reduced lactate levels in BC cells (Abooshahab et al., 2023[3][4]). This suggests that PEDF may help normalise cancer cell metabolism and potentiate the cytotoxic effects of Dox by limiting reliance on glycolysis, a crucial pathway for energy and biosynthesis in cancer cells.

The Pentose Phosphate Pathway (PPP), which branches from glycolysis at G6P, is crucial in managing oxidative stress by producing NADPH, a reducing equivalent necessary for regenerating antioxidants like glutathione (Perl et al., 2011[32], Ge et al., 2020[21]). In MDA-MB-231 cells, Dox increased G6P levels, indicating an activation of the PPP to counteract the oxidative stress induced by Dox (Songbo et al., 2019[39], Shi et al., 2023[37]). The increased PPP activity would provide NADPH, which is essential for neutralising reactive oxygen species (ROS) and maintaining cellular redox balance (Patra et al., 2014[31]). This adaptation highlights the metabolic flexibility of MDA-MB-231 cells, enabling them to redirect glucose metabolism into the PPP for antioxidant defense rather than energy production. Interestingly, when Dox is combined with PEDF, G6P intensity was decreased in MDA-MB-231 cells, which supports our previous research regarding the effect of PEDF on the levels of G6P in this cell line (Abooshahab et al., 2023[3]). This reduction suggests that PEDF may inhibit PPP activity, thereby limiting NADPH production and, consequently, the cell's capacity to counter oxidative stress, which can support the idea that PEDF can inhibit the generation of ROS (Yamagishi et al., 2005[44]). G6P was not identified in MCF-7 cells, potentially due to reduced dependency on the PPP for redox balance.

Amino acids are building blocks for protein synthesis and contribute to biosynthetic and antioxidant pathways in cancer cells (Chen et al., 2024[14]). Cysteine is essential for glutathione synthesis, a major cellular antioxidant (Chiang et al., 2022[15]). A study by Tacar et al. highlighted Dox's role in oxidative stress and its impact on cellular components, including thiol-containing molecules like cysteine (Tacar et al., 2013[41]). Additionally, research by Mehra et al. indicates that Dox-induced cardiomyopathy is associated with increased activity of cysteine proteases, suggesting alterations in cysteine metabolism (Mehra et al., 2017[30]). Our findings align with those observations, showing that in MDA-MB-231, Dox decreases cysteine intensity while increasing cystine in MCF-7, reflecting its consumption to combat ROS. The further depletion of cysteine with Dox+PEDF suggests PEDF may impair cysteine uptake or recycling, potentially limiting glutathione synthesis and weakening cellular antioxidant defences, thereby increasing susceptibility to apoptosis. The decrease in cysteine levels in MDA-MB-231 and the slight decrease in cystine levels in MCF-7 after combination exposure further corroborate the idea that a strong link exists between the perturbation in GSH metabolism and PEDF functions that may influence Dox activity in this area as well. 

Other amino acids, including asparagine, aspartic acid, glutamic acid, glycine, lysine, and proline, display differential responses to the treatments, suggesting disruptions in amino acid metabolism and its link to biosynthetic and redox homeostasis. Glutamic acid decreases significantly with Dox in both cell lines, especially in MDA-MB-231 cells. Glutamic acid is a critical amino acid for maintaining cellular redox balance, as it serves as a precursor for glutathione synthesis (Altman et al., 2016[8], Yoo et al., 2020[46]). The reduction of glutamic acid suggests an increased demand for glutathione synthesis to combat Dox-induced oxidative stress. However, with PEDF's presence, glutamic acid levels increased in MDA-MB-231 cells but remained low in MCF-7. PEDF's contrasting effects on glutamic acid levels between the two cell lines may indicate differences in how each cell line metabolises glutamate or regulates oxidative stress. In MDA-MB-231 cells, PEDF might protect against oxidative stress differently, perhaps by directly modulating ROS production, thereby lessening the need for glutathione synthesis. However, in MCF-7 cells, PEDF's effect could be less supportive in this way. 

The observed decrease in aspartic acid and uracil in MDA-MB-231 cells under Dox+PEDF treatment suggests a significant compromise in nucleotide synthesis, which is crucial for DNA repair and replication. Given Dox's mechanism of inducing DNA damage (Tacar et al., 2013[41]), disruption in nucleotide availability likely exacerbates the cytotoxic effects as cells struggle to repair damaged DNA effectively. In contrast, MCF-7 cells showed a reduction in asparagine, along with increased levels of aspartic acid and uracil, indicating a distinct impact on nucleotide synthesis in this cell line. However, since MCF-7 cells have a slower proliferation rate than MDA-MB-231 cells (Theodossiou et al., 2019[42]), this disruption in nucleotide synthesis may have less immediate consequences. Notwithstanding, limited nucleotide availability under Dox+PEDF treatment restricts MCF-7 cells' capacity for DNA repair, thereby enhancing Dox's cytotoxicity by further weakening cellular defences against DNA damage.

The regulation of lipid metabolism observed in both cell lines is noteworthy. Lipid metabolism, particularly fatty acid synthesis and cholesterol metabolism, is critical for rapidly-dividing cancer cells (Snaebjornsson et al., 2020[38], Fu et al., 2021[20]). Fatty acids are essential for membrane synthesis, energy storage, and signaling molecule production (Snaebjornsson et al., 2020[38]). Cholesterol plays a crucial role in maintaining membrane fluidity and regulating cellular signaling (Fu et al., 2021[20]). In our study, Dox+PEDF significantly impacts cholesterol metabolism in both cell lines, but with notable differences in scope and effect. Studies have demonstrated that Dox can suppress the expression of HMG-CoA reductase (HMGCR), a pivotal enzyme in cholesterol biosynthesis. This suppression results in reduced cholesterol levels, which is linked to the inhibition of the epidermal growth factor receptor (EGFR)-Src signaling pathway (Yan et al., 2020[45]). Research indicates that PEDF influences lipid metabolism, particularly by promoting lipolysis, the breakdown of fats into free fatty acids. This effect is mediated through its interaction with adipose triglyceride lipase (ATGL), an enzyme crucial for lipid metabolism (Borg et al., 2011[12]). However, its direct effect on cholesterol is not fully understood. Both PEDF and Dox independently reduced cholesterol levels in both cell lines, consistent with previous studies demonstrating their cholesterol-lowering effects (Yan et al., 2020[45], Abooshahab et al., 2023[3][4]). However, the combination led to a slight increase and decrease in MDA-MB-231 and MCF-7 cells, respectively, indicating cell line-specific responses to the combined treatment.

## 5. Conclusions

This study is a primary step towards establishing a robust platform for using in vitro metabolomics to gain a better picture of the biochemical alterations resulting from combined PEDF and Dox treatment. The combined effects of Dox and PEDF lead to a multifaceted disruption of metabolic pathways, synergistically and antagonistically targeting the cancer cells' metabolic adaptability. In summary, Dox and PEDF disrupt key metabolic pathways essential for cancer cell survival, including glycolysis, the PPP, amino acid, and lipid metabolism, underscoring their ability to hinder cancer cell energetics, antioxidant defenses, and biosynthetic capacity. PEDF was shown to normalise metabolic activity by reducing glycolysis reliance in both MDA-MB-231 and MCF-7 cell lines, thereby limiting lactate production and its associated tumour-promoting effects, either alone or in combination with Dox. Additionally, PEDF suppressed PPP activity by reducing G6P levels, impairing NADPH production and cellular antioxidant defenses, especially in MDA-MB-231 cells. This metabolic alteration enhances oxidative stress and reinforces Dox's cytotoxic effects.

The combination treatment also impacted amino acid metabolism, with significant depletion of cysteine, which weakens glutathione synthesis and antioxidant capacity, and may lead to heightened susceptibility to apoptosis. Furthermore, disruptions in glutamic acid, aspartic acid, and nucleotide synthesis pathways suggest impaired DNA repair and redox balance, contributing to the synergistic cytotoxicity of PEDF and Dox. The modulation of lipid metabolism, particularly cholesterol synthesis, by combination therapy further emphasises its potential to target key biosynthetic pathways critical for cancer cell proliferation and survival. Overall, the synergistic interaction between PEDF and Dox offers a promising therapeutic strategy that targets multiple metabolic vulnerabilities in BC cells. However, the distinct responses in different cell lines underscore the importance of tailoring treatments based on tumour-specific metabolic profiles for optimal efficacy. Further studies are warranted to investigate the translational potential of these findings, particularly in vivo settings and targeted analysis, to confirm the therapeutic relevance of PEDF and Dox combination therapy in BC treatment.

## Declaration

### Author Contributions:

Conceptualisation, CRD.; methodology, RA.; formal analysis, RA.; writing-original draft preparation, RA.; writing-review and editing, CRD, HAS.; supervision, CRD.; project administration CRD. All authors have read and agreed to the published version of the manuscript.

### Funding:

This research received no external funding.

### Informed Consent Statement:

Not applicable.

### Acknowledgments:

RA is sponsored by a Curtin University RTP HDR Scholarship.

### Conflicts of Interest: 

The authors declare no conflict of interest.

### Artificial Intelligence (AI) - Assisted Technology:

None was used in any stage of this work. 

### Data Availability Statement:

All data used and/or analyzed during the current study may be available from the corresponding author on reasonable request.

## Supplementary Material

Suppl. information

## Figures and Tables

**Table 1:**
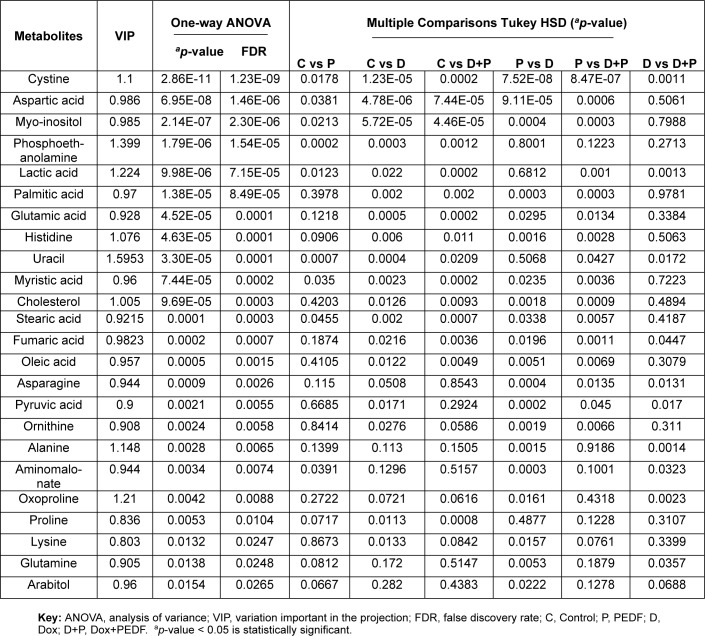
Significant alterations in MCF-7 metabolites.

**Table 2:**
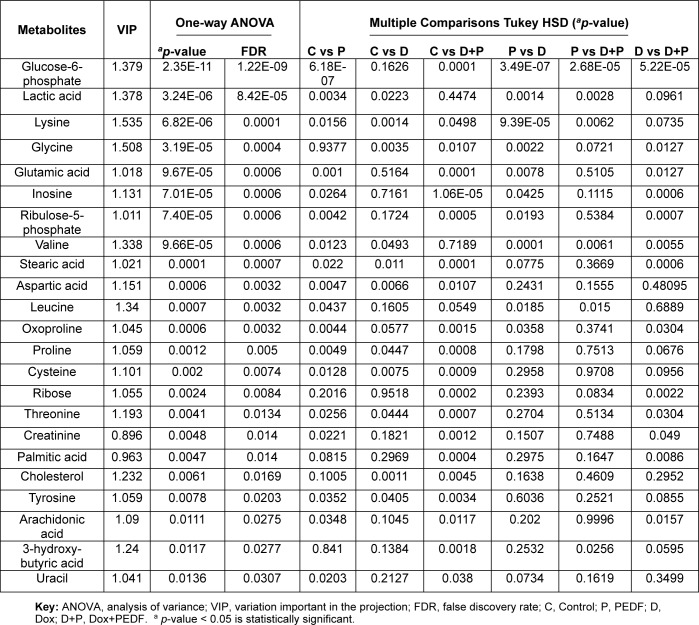
Significant alterations in MDA-MB-231 metabolites

**Figure 1:**
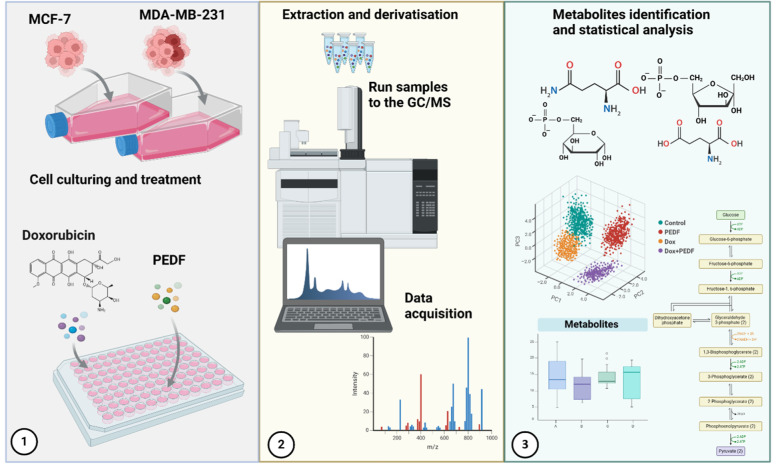
Graphical abstract
