# Supplementary material for: Targeting metabolic vulnerabilities in breast cancer cells by combining PEDF and doxorubicin: pathway insights from GC/MS-based metabolomics
Source: EXCLI J. 2025 Aug 18;24:1037–55. doi: 10.17179/excli2025-8508 (PMC12436679; doi:10.17179/excli2025-8508)
Supplement: Suppl. information [file EXCLI-24-1037-s-001.pdf]

EXCLI Journal 2025;24:1037-1055 – ISSN 1611-2156
Received: May 23, 2025, accepted: June 12, 2025, published: August 18, 2025

Supplementary information to:

Original article:

TARGETING METABOLIC VULNERABILITIES IN BREAST CANCER CELLS BY COMBINING PEDF AND DOXORUBICIN: PATHWAY INSIGHTS FROM GC/MS-BASED METABOLOMICS

Raziyeh Abooshahab[1,2], Hani Al-Salami[1,3], Crispin R. Dass[1,2,4*]

1 Curtin Medical School, Curtin University, Bentley 6102, Australia
2 Curtin Health Innovation Research Institute, Bentley 6102, Australia
3 Biotechnology and Drug Development Research Laboratory, Curtin Health Innovation Research Institute, Bentley 6102, Australia
4 Silpakorn University, Nakhon Pathom 73000, Thailand.

* **Corresponding author**: Crispin R. Dass (PhD), Professor, Curtin Medical School, Curtin University, Bentley 6102, Australia. Office: Curtin Health Innovation Research Institute, Bldg 305, Room 124, Curtin University, Bentley campus. Phone: +61 8 9266 1489
E-mail: Crispin.Dass@curtin.edu.au

https://dx.doi.org/10.17179/excli2025-8508

This is an Open Access article distributed under the terms of the Creative Commons Attribution License (http://creativecommons.org/licenses/by/4.0/)

EXCLI Journal 2025;24:1037-1055 – ISSN 1611-2156
Received: May 23, 2025, accepted: June 12, 2025, published: August 18, 2025

Table S1: The MS-DIAL parameters

**Data collection**	Mass range begin	50	Da
	Mass range end	600	Da
	Retention time begin	5.2	Min
	Retention time end	20	Min
	Number of threads	1	-
**Peak detection**	Minimum peak height	1000	-
	Smoothing method	Savitzky-Golay filter	-
**Deconvolution parameter**	Sigma window value	0.5	-
	EI abundance cut-off	10	-
**Identification**	RT tolerance	0.5	Min
	m/z tolerance	0.5	Da
	Identification score cut off	70%	-

EXCLI Journal 2025;24:1037-1055 – ISSN 1611-2156
Received: May 23, 2025, accepted: June 12, 2025, published: August 18, 2025

Table S2. Classification of MCF-7 metabolites formed from the ClassyFire system

InChIKey	Kingdom	Superclass	Class	Subclass	Parent Level 1	Parent Level 2	Parent Level 3	Parent Level 4
HVYWMOMLDIMFJA-DPAQBDIFSA-N	Organic compounds	Lipids and lipid-like molecules	Steroids and steroid derivatives	Cholestane steroids	Cholesterols and derivatives			
VBICKXHEKHSIBG-UHFFFAOYSA-N	Organic compounds	Lipids and lipid-like molecules	Glycerolipids	Monoradylglycerols	Monoacylglycerols	1-monoacylglycerols		
WHBMMWSBFZVSSR-GSVOUGTGSA-N	Organic compounds	Organic acids and derivatives	Hydroxy acids and derivatives	Beta hydroxy acids and derivatives	Beta hydroxy acids and derivatives			
QNAYBMKLOCPYGJ-REOHCLBHSA-N	Organic compounds	Organic acids and derivatives	Carboxylic acids and derivatives	Amino acids, peptides, and analogues	Amino acids and derivatives	Alpha amino acids and derivatives	Alanine and derivatives	
JINBYESILADKFW-UHFFFAOYSA-N	Organic compounds	Organic acids and derivatives	Carboxylic acids and derivatives	Amino acids, peptides, and analogues	Amino acids and derivatives	Alpha amino acids and derivatives	Alpha amino acids	
HEBKCHPVOIAQTA-QWWZWVQMSA-N	Organic compounds	Organic oxygen compounds	Organooxygen compounds	Carbohydrates and carbohydrate conjugates	Sugar alcohols			
DCXYFEDJOCDNAF-REOHCLBHSA-N	Organic compounds	Organic acids and derivatives	Carboxylic acids and derivatives	Amino acids, peptides, and analogues	Amino acids and derivatives	Alpha amino acids and derivatives	Asparagine and derivatives	
CKLJMWTZIZZHCS-REOHCLBHSA-N	Organic compounds	Organic acids and derivatives	Carboxylic acids and derivatives	Amino acids, peptides, and analogues	Amino acids and derivatives	Alpha amino acids and derivatives	Aspartic acid and derivatives	
UCMIRNVEIXFBKS-UHFFFAOYSA-N	Organic compounds	Organic acids and derivatives	Carboxylic acids and derivatives	Amino acids, peptides, and analogues	Amino acids and derivatives	Beta amino acids and derivatives		
KRKNYBCHXYNGOX-UHFFFAOYSA-N	Organic compounds	Organic acids and derivatives	Carboxylic acids and derivatives	Tricarboxylic acids and derivatives	Tricarboxylic acids and derivatives			
XUJNEKJLAYXESH-REOHCLBHSA-N	Organic compounds	Organic acids and derivatives	Carboxylic acids and derivatives	Amino acids, peptides, and analogues	Amino acids and derivatives	Alpha amino acids and derivatives	Cysteine and derivatives	

EXCLI Journal 2025;24:1037-1055 – ISSN 1611-2156
Received: May 23, 2025, accepted: June 12, 2025, published: August 18, 2025

InChIKey	Kingdom	Superclass	Class	Subclass	Parent Level 1	Parent Level 2	Parent Level 3	
LEVWYRKDKASIDU-UHFFFAOYSA-N	Organic compounds	Organic acids and derivatives	Carboxylic acids and derivatives	Amino acids, peptides, and analogues	Amino acids and derivatives	Alpha amino acids and derivatives	Cysteine and derivatives	
WHUUTDBJXJRKMK-VKHMYHEASA-N	Organic compounds	Organic acids and derivatives	Carboxylic acids and derivatives	Amino acids, peptides, and analogues	Amino acids and derivatives	Alpha amino acids and derivatives	Glutamic acid and derivatives	
ZDXPYRJPNDTMRX-VKHMYHEASA-N	Organic compounds	Organic acids and derivatives	Carboxylic acids and derivatives	Amino acids, peptides, and analogues	Amino acids and derivatives	Alpha amino acids and derivatives	Alpha amino acids	L-alpha-amino acids
PEDCQBHIVMGVHV-UHFFFAOYSA-N	Organic compounds	Organic oxygen compounds	Organooxygen compounds	Carbohydrates and carbohydrate conjugates	Sugar alcohols			
DHMQDGOQFOQNFH-UHFFFAOYSA-N	Organic compounds	Organic acids and derivatives	Carboxylic acids and derivatives	Amino acids, peptides, and analogues	Amino acids and derivatives	Alpha amino acids and derivatives	Alpha amino acids	
HNDVDQJCIGZPNO-YFKPBYRVSA-N	Organic compounds	Organic acids and derivatives	Carboxylic acids and derivatives	Amino acids, peptides, and analogues	Amino acids and derivatives	Alpha amino acids and derivatives	Histidine and derivatives	
INAPMGSXUVUWAF-GFWFORPUSA-N	Organic compounds	Organic oxygen compounds	Organooxygen compounds	Alcohols and polyols	Cyclic alcohols and derivatives	Cyclitols and derivatives	Inositol phosphates	
AGPKZVBTJJNPAG-WHFBIAKZSA-N	Organic compounds	Organic acids and derivatives	Carboxylic acids and derivatives	Amino acids, peptides, and analogues	Amino acids and derivatives	Alpha amino acids and derivatives	Isoleucine and derivatives	
JVTAAEKCZFNVCJ-UHFFFAOYSA-N	Organic compounds	Organic acids and derivatives	Hydroxy acids and derivatives	Alpha hydroxy acids and derivatives	Alpha hydroxy acids and derivatives			
ROHFNLRQFUQHCH-YFKPBYRVSA-N	Organic compounds	Organic acids and derivatives	Carboxylic acids and derivatives	Amino acids, peptides, and analogues	Amino acids and derivatives	Alpha amino acids and derivatives	Leucine and derivatives	
KDXKERNSBIXSRK-YFKPBYRVSA-N	Organic compounds	Organic acids and derivatives	Carboxylic acids and derivatives	Amino acids, peptides, and analogues	Amino acids and derivatives	Alpha amino acids and derivatives	Alpha amino acids	L-alpha-amino acids
BJEPYKJPYRNKOW-UHFFFAOYSA-N	Organic compounds	Organic acids and derivatives	Hydroxy acids and derivatives	Beta hydroxy acids and derivatives	Beta hydroxy acids and derivatives			

EXCLI Journal 2025;24:1037-1055 – ISSN 1611-2156
Received: May 23, 2025, accepted: June 12, 2025, published: August 18, 2025

InChIKey	Kingdom	Superclass	Class	Subclass	Parent Level 1	Parent Level 2	Parent Level 3	Parent Level 4
FYGDTMLNYKFZSV-DZOUCCHMSA-N	Organic compounds	Organic oxygen compounds	Organooxygen compounds	Carbohydrates and carbohydrate conjugates	Oligosaccharides			
FYGDTMLNYKFZSV-DZOUCCHMSA-N	Organic compounds	Organic oxygen compounds	Organooxygen compounds	Carbohydrates and carbohydrate conjugates	Oligosaccharides			
FFEARJCKVFRZRR-BYPYZUCNSA-N	Organic compounds	Organic acids and derivatives	Carboxylic acids and derivatives	Amino acids, peptides, and analogues	Amino acids and derivatives	Alpha amino acids and derivatives	Methionine and derivatives	
TUNFSRHWOTWDNC-UHFFFAOYSA-N	Organic compounds	Lipids and lipid-like molecules	Fatty Acyls	Fatty acids and conjugates	Long-chain fatty acids			
ZQPPMHVWECSIRJ-KTKRTIGZSA-N	Organic compounds	Lipids and lipid-like molecules	Fatty Acyls	Fatty acids and conjugates	Long-chain fatty acids			
AHLPHDHHMVZTML-BYPYZUCNSA-N	Organic compounds	Organic acids and derivatives	Carboxylic acids and derivatives	Amino acids, peptides, and analogues	Amino acids and derivatives	Alpha amino acids and derivatives	Alpha amino acids	L-alpha-amino acids
ODHCTXKNWHHXJC-VKHMYHEASA-N	Organic compounds	Organic acids and derivatives	Carboxylic acids and derivatives	Amino acids, peptides, and analogues	Amino acids and derivatives	Alpha amino acids and derivatives		
IPCSVZSSVZVIGE-UHFFFAOYSA-N	Organic compounds	Lipids and lipid-like molecules	Fatty Acyls	Fatty acids and conjugates	Long-chain fatty acids			
COLNVLDHVKWLRT-QMMMGPOBSA-N	Organic compounds	Organic acids and derivatives	Carboxylic acids and derivatives	Amino acids, peptides, and analogues	Amino acids and derivatives	Alpha amino acids and derivatives	Phenylalanine and derivatives	
SUHOOTKUPISOBE-UHFFFAOYSA-N	Organic compounds	Organic acids and derivatives	Organic phosphoric acids and derivatives	Phosphate esters	Phosphoethanolamines			
ONIBWKKTOPOVIA-BYPYZUCNSA-N	Organic compounds	Organic acids and derivatives	Carboxylic acids and derivatives	Amino acids, peptides, and analogues	Amino acids and derivatives	Alpha amino acids and derivatives	Proline and derivatives	

EXCLI Journal 2025;24:1037-1055 – ISSN 1611-2156
Received: May 23, 2025, accepted: June 12, 2025, published: August 18, 2025

InChIKey	Kingdom	Superclass	Class	Subclass	Parent Level 1		Parent Level 2	Parent Level 3
LCTONWCANYUPML-UHFFFAOYSA-N	Organic compounds	Organic acids and derivatives	Keto acids and derivatives	Alpha-keto acids and derivatives	Alpha-keto acids and derivatives			
MTCFGRXMJLQNBG-REOHCLBHSA-N	Organic compounds	Organic acids and derivatives	Carboxylic acids and derivatives	Amino acids, peptides, and analogues	Amino acids and derivatives	Alpha amino acids and derivatives	Serine and derivatives	
QIQXTHQIDYTFRH-UHFFFAOYSA-N	Organic compounds	Lipids and lipid-like molecules	Fatty Acyls	Fatty acids and conjugates	Long-chain fatty acids			
KDYFGRWQOYBRFD-UHFFFAOYSA-N	Organic compounds	Organic acids and derivatives	Carboxylic acids and derivatives	Dicarboxylic acids and derivatives	Dicarboxylic acids and derivatives			
AYFVYJQAPQTCCC-GBXIJSLDSA-N	Organic compounds	Organic acids and derivatives	Carboxylic acids and derivatives	Amino acids, peptides, and analogues	Amino acids and derivatives	Alpha amino acids and derivatives	Alpha amino acids	L-alpha-amino acids
QIVBCDIJIAJPQS-VIFPVBQESA-N	Organic compounds	Organoheterocyclic compounds	Indoles and derivatives	Indolyl carboxylic acids and derivatives	Indolyl carboxylic acids and derivatives			
OUYCCCASQSFEME-QMMMGPOBSA-N	Organic compounds	Organic acids and derivatives	Carboxylic acids and derivatives	Amino acids, peptides, and analogues	Amino acids and derivatives	Alpha amino acids and derivatives	Tyrosine and derivatives	
ISAKRJDGNUQOIC-UHFFFAOYSA-N	Organic compounds	Organoheterocyclic compounds	Diazines	Pyrimidines and pyrimidine derivatives	Pyrimidones			
KZSNJWFQEVHDMF-BYPYZUCNSA-N	Organic compounds	Organic acids and derivatives	Carboxylic acids and derivatives	Amino acids, peptides, and analogues	Amino acids and derivatives	Alpha amino acids and derivatives	Valine and derivatives	

EXCLI Journal 2025;24:1037-1055 – ISSN 1611-2156
Received: May 23, 2025, accepted: June 12, 2025, published: August 18, 2025

Table S3: Classification of MDA-MB231 metabolites formed from the ClassyFire system

InChIKey	Kingdom	Superclass	Class	Subclass	Parent Level 1	Parent Level 2	Parent Level 3	Parent Level 4
HVYWMOMLDIMFJA-DPAQBDIFSA-N	Organic compounds	Lipids and lipid-like molecules	Steroids and steroid derivatives	Cholestane steroids	Cholesterols and derivatives			
QHZLMUACJMDIAE-UHFFFAOYSA-N	Organic compounds	Lipids and lipid-like molecules	Glycerolipids	Monoradylglycerols	Monoacylglycerols	1-monoacylglycerols		
WHBMMWSBFZVSSR-GSVOUGTGSA-N	Organic compounds	Organic acids and derivatives	Hydroxy acids and derivatives	Beta hydroxy acids and derivatives	Beta hydroxy acids and derivatives			
OSJPPGNTCRNQQC-UHFFFAOYSA-N	Organic compounds	Organic oxygen compounds	Organooxygen compounds	Carbohydrates and carbohydrate conjugates	Sugar acids and derivatives			
BTCSSZJGUNDROE-UHFFFAOYSA-N	Organic compounds	Organic acids and derivatives	Carboxylic acids and derivatives	Amino acids, peptides, and analogues	Amino acids and derivatives	Gamma amino acids and derivatives		
OIRDTQYFTABQOQ-KQYNXXCUSA-N	Organic compounds	Nucleosides, nucleotides, and analogues	Purine nucleosides		Purine nucleosides			
UDMBCSSLTHHNCD-KQYNXXCUSA-N	Organic compounds	Nucleosides, nucleotides, and analogues	Purine nucleotides	Purine ribonucleotides	Purine ribonucleoside monophosphates			
QNAYBMKLOCPYGJ-REOHCLBHSA-N	Organic compounds	Organic acids and derivatives	Carboxylic acids and derivatives	Amino acids, peptides, and analogues	Amino acids and derivatives	Alpha amino acids and derivatives	Alanine and derivatives	
KPGXRSRHYNQIFN-UHFFFAOYSA-N	Organic compounds	Organic acids and derivatives	Keto acids and derivatives	Gamma-keto acids and derivatives	Gamma-keto acids and derivatives			
YZXBAPSDXZZRGB-DOFZRALJSA-N	Organic compounds	Lipids and lipid-like molecules	Fatty Acyls	Fatty acids and conjugates	Long-chain fatty acids			
DCXYFEDJOCDNAF-REOHCLBHSA-N	Organic compounds	Organic acids and derivatives	Carboxylic acids and derivatives	Amino acids, peptides, and analogues	Amino acids and derivatives	Alpha amino acids and derivatives	Asparagine and derivatives	

EXCLI Journal 2025;24:1037-1055 – ISSN 1611-2156
Received: May 23, 2025, accepted: June 12, 2025, published: August 18, 2025

InChIKey	Kingdom	Superclass	Class	Subclass	Parent Level 1	Parent Level 2	Parent Level 3	Parent Level 4
CKLJMWTZIZZHCS-REOHCLBHSA-N	Organic compounds	Organic acids and derivatives	Carboxylic acids and derivatives	Amino acids, peptides, and analogues	Amino acids and derivatives	Alpha amino acids and derivatives	Aspartic acid and derivatives	
UCMIRN-VEIXFBKS-UHFFFAOYSA-N	Organic compounds	Organic acids and derivatives	Carboxylic acids and derivatives	Amino acids, peptides, and analogues	Amino acids and derivatives	Beta amino acids and derivatives		
KRKNYB-CHXYNGOX-UHFFFAOYSA-N	Organic compounds	Organic acids and derivatives	Carboxylic acids and derivatives	Tricarboxylic acids and derivatives	Tricarboxylic acids and derivatives			
DDRJAANPRJI-HGJ-UHFFFAOYSA-N	Organic compounds	Organic acids and derivatives	Carboxylic acids and derivatives	Amino acids, peptides, and analogues	Amino acids and derivatives	Alpha amino acids and derivatives		
XUJNEKJLAYX-ESH-REO-HCLBHSA-N	Organic compounds	Organic acids and derivatives	Carboxylic acids and derivatives	Amino acids, peptides, and analogues	Amino acids and derivatives	Alpha amino acids and derivatives	Cysteine and derivatives	
NBSCHQHZLSJFNQ-VFUOTHLCSA-N	Organic compounds	Organic oxygen compounds	Organooxygen compounds	Carbohydrates and carbohydrate conjugates	Monosaccharides	Hexoses	Hexose phosphates	
WHUUTDBJXJRKMK-VKH-MYHEASA-N	Organic compounds	Organic acids and derivatives	Carboxylic acids and derivatives	Amino acids, peptides, and analogues	Amino acids and derivatives	Alpha amino acids and derivatives	Glutamic acid and derivatives	
ZDXPYRJPNDTMRX-VKHMYHEASA-N	Organic compounds	Organic acids and derivatives	Carboxylic acids and derivatives	Amino acids, peptides, and analogues	Amino acids and derivatives	Alpha amino acids and derivatives	Alpha amino acids	L-alpha-amino acids
JFCQEDHGNNZCLN-UHFFFAOYSA-N	Organic compounds	Organic acids and derivatives	Carboxylic acids and derivatives	Dicarboxylic acids and derivatives	Dicarboxylic acids and derivatives			
PEDCQBHIVMGVHV-UHFFFAOYSA-N	Organic compounds	Organic oxygen compounds	Organooxygen compounds	Carbohydrates and carbohydrate conjugates	Sugar alcohols			
DHMQDGOQFOQNFH-UHFFFAOYSA-N	Organic compounds	Organic acids and derivatives	Carboxylic acids and derivatives	Amino acids, peptides, and analogues	Amino acids and derivatives	Alpha amino acids and derivatives	Alpha amino acids	

InChIKey	Kingdom	Superclass	Class	Subclass	Parent Level 1	Parent Level 2	Parent Level 3	Parent Level 4
UGQMRVRMYYASKQ-KQYNXXCUSA-N	Organic compounds	Nucleosides, nucleotides, and analogues	Purine nucleosides		Purine nucleosides			
JVTAAEKCZFNVCJ-UHFFFAOYSA-N	Organic compounds	Organic acids and derivatives	Hydroxy acids and derivatives	Alpha hydroxy acids and derivatives	Alpha hydroxy acids and derivatives			
ROHFNLRQFUQHCH-YFKPBYRVSA-N	Organic compounds	Organic acids and derivatives	Carboxylic acids and derivatives	Amino acids, peptides, and analogues	Amino acids and derivatives	Alpha amino acids and derivatives	Leucine and derivatives	
AGPKZVBTJJNPAG-WHFBIAKZSA-N	Organic compounds	Organic acids and derivatives	Carboxylic acids and derivatives	Amino acids, peptides, and analogues	Amino acids and derivatives	Alpha amino acids and derivatives	Isoleucine and derivatives	
KDXKERNSBIXSRK-YFKPBYRVSA-N	Organic compounds	Organic acids and derivatives	Carboxylic acids and derivatives	Amino acids, peptides, and analogues	Amino acids and derivatives	Alpha amino acids and derivatives	Alpha amino acids	L-alpha-amino acids
BJEPYKJPYRNKOW-UHFFFAOYSA-N	Organic compounds	Organic acids and derivatives	Hydroxy acids and derivatives	Beta hydroxy acids and derivatives	Beta hydroxy acids and derivatives			
FFEARJCKVFRZRR-BYPYZUCNSA-N	Organic compounds	Organic acids and derivatives	Carboxylic acids and derivatives	Amino acids, peptides, and analogues	Amino acids and derivatives	Alpha amino acids and derivatives	Methionine and derivatives	
ZQPPMHVWECSIRJ-KTKRTIGZSA-N	Organic compounds	Lipids and lipid-like molecules	Fatty Acyls	Fatty acids and conjugates	Long-chain fatty acids			
ODHCTXKNWHHXJC-VKHMYHEASA-N	Organic compounds	Organic acids and derivatives	Carboxylic acids and derivatives	Amino acids, peptides, and analogues	Amino acids and derivatives	Alpha amino acids and derivatives		
IPCSVZSSVZVIGE-UHFFFAOYSA-N	Organic compounds	Lipids and lipid-like molecules	Fatty Acyls	Fatty acids and conjugates	Long-chain fatty acids			
COLNVLDHVKWLRT-QMMMGPOBSA-N	Organic compounds	Organic acids and derivatives	Carboxylic acids and derivatives	Amino acids, peptides, and analogues	Amino acids and derivatives	Alpha amino acids and derivatives	Phenylalanine and derivatives	

EXCLI Journal 2025;24:1037-1055 – ISSN 1611-2156
Received: May 23, 2025, accepted: June 12, 2025, published: August 18, 2025

InChIKey	Kingdom	Superclass	Class	Subclass	Parent Level 1	Parent Level 2	Parent Level 3	Parent Level 4
SUHOOTKUPISOBE-UHFFFAOYSA-N	Organic compounds	Organic acids and derivatives	Organic phosphoric acids and derivatives	Phosphate esters	Phosphoethanolamines			
ONIBWKKTOPOVIA-BYPYZUCNSA-N	Organic compounds	Organic acids and derivatives	Carboxylic acids and derivatives	Amino acids, peptides, and analogues	Amino acids and derivatives	Alpha amino acids and derivatives	Proline and derivatives	
HMFHBZSHGGEWLOSOOFDHNKSA-N	Organic compounds	Organic oxygen compounds	Organooxygen compounds	Carbohydrates and carbohydrate conjugates	Monosaccharides	Pentoses		
FNZLKVNUWIIPSJ-UHNVWZDZSA-N	Organic compounds	Organic oxygen compounds	Organooxygen compounds	Carbohydrates and carbohydrate conjugates	Monosaccharides	Pentoses	Pentose phosphates	
MTCFGRXMJLQNBG-REOHCLBHSA-N	Organic compounds	Organic acids and derivatives	Carboxylic acids and derivatives	Amino acids, peptides, and analogues	Amino acids and derivatives	Alpha amino acids and derivatives	Serine and derivatives	
QIQXTHQIDYTFRH-UHFFFAOYSA-N	Organic compounds	Lipids and lipid-like molecules	Fatty Acyls	Fatty acids and conjugates	Long-chain fatty acids			
AYFVYJQAPQTCCC-GBXIJSLDSA-N	Organic compounds	Organic acids and derivatives	Carboxylic acids and derivatives	Amino acids, peptides, and analogues	Amino acids and derivatives	Alpha amino acids and derivatives	Alpha amino acids	L-alpha-amino acids
QIVBCDIJIAJPQS-VIFPVBQESA-N	Organic compounds	Organoheterocyclic compounds	Indoles and derivatives	Indolyl carboxylic acids and derivatives	Indolyl carboxylic acids and derivatives			
OUYCCCASQSFEME-QMMMGPOBSA-N	Organic compounds	Organic acids and derivatives	Carboxylic acids and derivatives	Amino acids, peptides, and analogues	Amino acids and derivatives	Alpha amino acids and derivatives	Tyrosine and derivatives	
ISAKRJDGNUQOIC-UHFFFAOYSA-N	Organic compounds	Organoheterocyclic compounds	Diazines	Pyrimidines and pyrimidine derivatives	Pyrimidones			
KZSNJWFQEVHDMF-BYPYZUCNSA-N	Organic compounds	Organic acids and derivatives	Carboxylic acids and derivatives	Amino acids, peptides, and analogues	Amino acids and derivatives	Alpha amino acids and derivatives	Valine and derivatives	

EXCLI Journal 2025;24:1037-1055 – ISSN 1611-2156
Received: May 23, 2025, accepted: June 12, 2025, published: August 18, 2025

InChIKey	Kingdom	Superclass	Class	Subclass	Parent Level 1	Parent Level 2	Parent Level 3	Parent Level 4
LCTONWCANYUPML-UHFFFAOYSA-N	Organic compounds	Organic acids and derivatives	Keto acids and derivatives	Alpha-keto acids and derivatives	Alpha-keto acids and derivatives			
JINBYESILADKFW-UHFFFAOYSA-N	Organic compounds	Organic acids and derivatives	Carboxylic acids and derivatives	Amino acids, peptides, and analogues	Amino acids and derivatives	Alpha amino acids and derivatives	Alpha amino acids	
KDYFGRWQOYBRFD-UHFFFAOYSA-N	Organic compounds	Organic acids and derivatives	Carboxylic acids and derivatives	Dicarboxylic acids and derivatives	Dicarboxylic acids and derivatives			
BKAJNAXTPSGJCU-UHFFFAOYSA-N	Organic compounds	Organic acids and derivatives	Keto acids and derivatives	Short-chain keto acids and derivatives	Short-chain keto acids and derivatives			
LEVWYRKDKASIDU-IMJSIDKUSA-N	Organic compounds	Organic acids and derivatives	Carboxylic acids and derivatives	Amino acids, peptides, and analogues	Amino acids and derivatives	Alpha amino acids and derivatives	Cysteine and derivatives	L-cysteine-S-conjugates
DTBNBXWJWCWCIK-UHFFFAOYSA-N	Organic compounds	Organic acids and derivatives	Organic phosphoric acids and derivatives	Phosphate esters	Phosphate esters			

Table S4: Pathway Enrichment analysis of altered metabolites in MCF-7 between Control and PEDF

Metabolic Pathway	[a]Total Cmpd	[b]Hits	[c]Raw p	[d]FDR
Phosphatidylethanolamine Biosynthesis	12	1	0.0238	1
Phosphatidylcholine Biosynthesis	14	1	0.0278	1
Beta-Alanine Metabolism	34	1	0.0667	1
Sphingolipid Metabolism	40	1	0.0783	1
Pyrimidine Metabolism	57	1	0.111	1

[a] Total Cmpd: total number of compounds in the pathway, [b] Hit: actually matched number from the data
[c] Raw p: p-value calculated from the enrichment analysis, [d] FDR: p-value adjusted using False Discovery Rate

EXCLI Journal 2025;24:1037-1055 – ISSN 1611-2156
Received: May 23, 2025, accepted: June 12, 2025, published: August 18, 2025

Table S5: Pathway Enrichment analysis of altered metabolites in MCF-7 between Control and Dox

Metabolic Pathway	[a]Total Cmpd	[b]Hits	[c]Raw p	FDR
Urea Cycle	28	5	5.78E-05	0.00566
Beta-Alanine Metabolism	34	5	0.000154	0.00754
Arginine and Proline Metabolism	52	5	0.00119	0.0331
Ammonia Recycling	31	4	0.00135	0.0331
Aspartate Metabolism	35	4	0.00215	0.0422
Malate-Aspartate Shuttle	10	2	0.0113	0.18
Warburg Effect	57	4	0.0129	0.18
Glucose-Alanine Cycle	13	2	0.0189	0.232
Histidine Metabolism	42	3	0.0309	0.311
Alanine Metabolism	17	2	0.0317	0.311
Glutamate Metabolism	48	3	0.0437	0.389
Methylhistidine Metabolism	4	1	0.0663	0.485
Cysteine Metabolism	26	2	0.0694	0.485
Glycine and Serine Metabolism	59	3	0.0731	0.485
Phenylalanine and Tyrosine Metabolism	27	2	0.0742	0.485
Lysine Degradation	30	2	0.0892	0.537
Citric Acid Cycle	32	2	0.0997	0.537
Amino Sugar Metabolism	33	2	0.105	0.537
Gluconeogenesis	33	2	0.105	0.537
Tyrosine Metabolism	70	3	0.11	0.537
Fatty Acid Biosynthesis	35	2	0.116	0.538
Purine Metabolism	73	3	0.121	0.538
Biotin Metabolism	8	1	0.128	0.547
Propanoate Metabolism	42	2	0.157	0.619
Pyruvaldehyde Degradation	10	1	0.158	0.619
Phosphatidylethanolamine Biosynthesis	12	1	0.187	0.679
Pyruvate Metabolism	47	2	0.188	0.679
Steroid Biosynthesis	48	2	0.194	0.679
Phosphatidylcholine Biosynthesis	14	1	0.214	0.724
Pyrimidine Metabolism	57	2	0.251	0.804
Phosphatidylinositol Phosphate Metabolism	17	1	0.254	0.804
Spermidine and Spermine Biosynthesis	18	1	0.267	0.818
Mitochondrial Electron Transport Chain	19	1	0.28	0.831
Glutathione Metabolism	20	1	0.292	0.838
Bile Acid Biosynthesis	65	2	0.303	0.838
Carnitine Synthesis	22	1	0.316	0.838
Transfer of Acetyl Groups into Mitochondria	22	1	0.316	0.838
Glycolysis	23	1	0.328	0.847
Inositol Phosphate Metabolism	24	1	0.34	0.854
Glycerolipid Metabolism	25	1	0.351	0.861
Plasmalogen Synthesis	26	1	0.363	0.867

EXCLI Journal 2025;24:1037-1055 – ISSN 1611-2156
Received: May 23, 2025, accepted: June 12, 2025, published: August 18, 2025

Mitochondrial Beta-Oxidation of Long Chain Saturated Fatty Acids	28	1	0.385	0.898
Folate Metabolism	29	1	0.396	0.901
Inositol Metabolism	30	1	0.406	0.904
Nicotinate and Nicotinamide Metabolism	35	1	0.456	0.972
Fatty Acid Elongation In Mitochondria	35	1	0.456	0.972
Galactose Metabolism	38	1	0.485	1
Sphingolipid Metabolism	40	1	0.503	1
Fatty acid Metabolism	43	1	0.528	1
Steroidogenesis	43	1	0.528	1
Valine, Leucine and Isoleucine Degradation	59	1	0.647	1
Tryptophan Metabolism	59	1	0.647	1
Arachidonic Acid Metabolism	67	1	0.695	1

[a] Total Cmpd: total number of compounds in the pathway, [b] Hit: actually matched number from the data
[c] Raw p: p-value calculated from the enrichment analysis, [d] FDR: p-value adjusted using False Discovery Rate

Table S6: Pathway Enrichment analysis of altered metabolites in MCF-7 between Control and Dox+PEDF

Metabolic Pathway	[a]Total Cmpd	[b]Hits	[c]Raw p	[d]FDR
Arginine and Proline Metabolism	52	4	0.00235	0.113
Urea Cycle	28	3	0.00364	0.113
Ammonia Recycling	31	3	0.00489	0.113
Malate-Aspartate Shuttle	10	2	0.00561	0.113
Beta-Alanine Metabolism	34	3	0.00638	0.113
Aspartate Metabolism	35	3	0.00693	0.113
Warburg Effect	57	3	0.0266	0.373
Phenylalanine and Tyrosine Metabolism	27	2	0.0391	0.451
Tyrosine Metabolism	70	3	0.0455	0.451
Methylhistidine Metabolism	4	1	0.0471	0.451
Purine Metabolism	73	3	0.0507	0.451
Fatty Acid Biosynthesis	35	2	0.0628	0.513
Histidine Metabolism	42	2	0.0868	0.654
Steroid Biosynthesis	48	2	0.109	0.714
Glutamate Metabolism	48	2	0.109	0.714
Phosphatidylethanolamine Biosynthesis	12	1	0.135	0.829
Glucose-Alanine Cycle	13	1	0.146	0.84
Phosphatidylcholine Biosynthesis	14	1	0.156	0.85
Bile Acid Biosynthesis	65	2	0.18	0.871
Alanine Metabolism	17	1	0.187	0.871
Phosphatidylinositol Phosphate Metabolism	17	1	0.187	0.871
Mitochondrial Electron Transport Chain	19	1	0.206	0.919
Glutathione Metabolism	20	1	0.216	0.92
Inositol Phosphate Metabolism	24	1	0.254	0.95
Glycerolipid Metabolism	25	1	0.263	0.95
Cysteine Metabolism	26	1	0.272	0.95
Plasmalogen Synthesis	26	1	0.272	0.95
Mitochondrial Beta-Oxidation of Long Chain Saturated Fatty Acids	28	1	0.29	0.95
Folate Metabolism	29	1	0.298	0.95
Inositol Metabolism	30	1	0.307	0.95
Lysine Degradation	30	1	0.307	0.95
Citric Acid Cycle	32	1	0.324	0.95
Amino Sugar Metabolism	33	1	0.332	0.95
Gluconeogenesis	33	1	0.332	0.95
Nicotinate and Nicotinamide Metabolism	35	1	0.349	0.95
Fatty Acid Elongation In Mitochondria	35	1	0.349	0.95
Galactose Metabolism	38	1	0.373	0.982
Sphingolipid Metabolism	40	1	0.388	0.982
Propanoate Metabolism	42	1	0.404	0.982
Fatty acid Metabolism	43	1	0.411	0.982
Steroidogenesis	43	1	0.411	0.982
Pyruvate Metabolism	47	1	0.44	1

EXCLI Journal 2025;24:1037-1055 – ISSN 1611-2156
Received: May 23, 2025, accepted: June 12, 2025, published: August 18, 2025

Glycine and Serine Metabolism	59	1	0.519	1
Valine, Leucine and Isoleucine Degradation	59	1	0.519	1
Tryptophan Metabolism	59	1	0.519	1
Arachidonic Acid Metabolism	67	1	0.566	1

[a] Total Cmpd: total number of compounds in the pathway, [b] Hit: actually matched number from the data
[c] Raw p: p-value calculated from the enrichment analysis, [d] FDR: p-value adjusted using False Discovery Rate

EXCLI Journal 2025;24:1037-1055 – ISSN 1611-2156
Received: May 23, 2025, accepted: June 12, 2025, published: August 18, 2025

Table S7: Pathway Enrichment analysis of altered metabolites in MDA-MB231 between Control and PEDF

Metabolic Pathways	[a]Total Cmpd	[b]Hits	[c]Raw p	[d]FDR
Glutathione Metabolism	20	3	0.00134	0.131
Warburg Effect	57	4	0.00331	0.162
Malate-Aspartate Shuttle	10	2	0.00561	0.183
Glutamate Metabolism	48	3	0.0167	0.408
Arginine and Proline Metabolism	52	3	0.0208	0.408
Cysteine Metabolism	26	2	0.0364	0.474
Urea Cycle	28	2	0.0418	0.474
Pentose Phosphate Pathway	29	2	0.0446	0.474
Lysine Degradation	30	2	0.0475	0.474
Ammonia Recycling	31	2	0.0504	0.474
Gluconeogenesis	33	2	0.0565	0.474
Beta-Alanine Metabolism	34	2	0.0596	0.474
Aspartate Metabolism	35	2	0.0628	0.474
Galactose Metabolism	38	2	0.0728	0.509
Propanoate Metabolism	42	2	0.0868	0.565
Biotin Metabolism	8	1	0.0922	0.565
Homocysteine Degradation	9	1	0.103	0.595
Taurine and Hypotaurine Metabolism	12	1	0.135	0.719
Glucose-Alanine Cycle	13	1	0.146	0.719
Glycine and Serine Metabolism	59	2	0.154	0.719
Valine, Leucine and Isoleucine Degradation	59	2	0.154	0.719
Alanine Metabolism	17	1	0.187	0.831
Tyrosine Metabolism	70	2	0.202	0.847
Purine Metabolism	73	2	0.216	0.847
Nucleotide Sugars Metabolism	20	1	0.216	0.847
Pantothenate and CoA Biosynthesis	21	1	0.226	0.85
Carnitine Synthesis	22	1	0.235	0.853
Glycolysis	23	1	0.244	0.855
Inositol Phosphate Metabolism	24	1	0.254	0.857
Glycerolipid Metabolism	25	1	0.263	0.858
Plasmalogen Synthesis	26	1	0.272	0.859
Phenylalanine and Tyrosine Metabolism	27	1	0.281	0.859
Mitochondrial Beta-Oxidation of Long Chain Saturated Fatty Acids	28	1	0.29	0.859
Folate Metabolism	29	1	0.298	0.859
Inositol Metabolism	30	1	0.307	0.859
Starch and Sucrose Metabolism	31	1	0.316	0.859
Amino Sugar Metabolism	33	1	0.332	0.881
Nicotinate and Nicotinamide Metabolism	35	1	0.349	0.9
Methionine Metabolism	42	1	0.404	0.989
Histidine Metabolism	42	1	0.404	0.989
Pyruvate Metabolism	47	1	0.44	1

EXCLI Journal 2025;24:1037-1055 – ISSN 1611-2156
Received: May 23, 2025, accepted: June 12, 2025, published: August 18, 2025

Tryptophan Metabolism	59	1	0.519	1
Arachidonic Acid Metabolism	67	1	0.566	1

[a] Total Cmpd: total number of compounds in the pathway, [b] Hit: actually matched number from the data [c] Raw p: p-value calculated from the enrichment analysis, [d] FDR: p-value adjusted using False Discovery Rate

EXCLI Journal 2025;24:1037-1055 – ISSN 1611-2156
Received: May 23, 2025, accepted: June 12, 2025, published: August 18, 2025

Table S8: Pathway Enrichment analysis of altered metabolites in MDA-MB231 between Control and Dox

Metabolic Pathways	[a]Total Cmpd	[b]Hits	[c]Raw p	[d]FDR
Carnitine Synthesis	22	2	0.00269	0.262
Ammonia Recycling	31	2	0.00535	0.262
Glutamate Metabolism	48	2	0.0127	0.363
Arginine and Proline Metabolism	52	2	0.0148	0.363
Bile Acid Biosynthesis	65	2	0.0228	0.442
Purine Metabolism	73	2	0.0285	0.442
Biotin Metabolism	8	1	0.0316	0.442
Malate-Aspartate Shuttle	10	1	0.0394	0.482
Alanine Metabolism	17	1	0.0663	0.721
Glutathione Metabolism	20	1	0.0776	0.76
Urea Cycle	28	1	0.107	0.929
Lysine Degradation	30	1	0.115	0.929
Beta-Alanine Metabolism	34	1	0.129	0.929
Aspartate Metabolism	35	1	0.133	0.929
Porphyrin Metabolism	40	1	0.151	0.929
Methionine Metabolism	42	1	0.158	0.929
Steroidogenesis	43	1	0.161	0.929
Steroid Biosynthesis	48	1	0.179	0.972
Glycine and Serine Metabolism	59	1	0.216	1
Tyrosine Metabolism	70	1	0.252	1

[a] Total Cmpd: total number of compounds in the pathway, [b] Hit: actually matched number from the data
[c] Raw p: p-value calculated from the enrichment analysis, [d] FDR: p-value adjusted using False Discovery Rate

Table S9: Pathway Enrichment analysis of altered metabolites in MDA-MB231 between Control and Dox+PEDF

Metabolic Pathways	[a]Total Cmpd	Hits	Raw p	FDR
Glutamate Metabolism	48	6	0.000205	0.0201
Glutathione Metabolism	20	4	0.000457	0.0214
Glycine and Serine Metabolism	59	6	0.000657	0.0214
Ammonia Recycling	31	4	0.00257	0.0631
Alanine Metabolism	17	3	0.00387	0.0759
Cysteine Metabolism	26	3	0.0132	0.175
Malate-Aspartate Shuttle	10	2	0.0155	0.175
Urea Cycle	28	3	0.0162	0.175
Arginine and Proline Metabolism	52	4	0.0168	0.175
Pentose Phosphate Pathway	29	3	0.0179	0.175
Warburg Effect	57	4	0.023	0.205
Glucose-Alanine Cycle	13	2	0.0259	0.211
Methionine Metabolism	42	3	0.0475	0.358
Purine Metabolism	73	4	0.0515	0.36
Glycolysis	23	2	0.0745	0.487
Phenylalanine and Tyrosine Metabolism	27	2	0.0986	0.604
Bile Acid Biosynthesis	65	3	0.135	0.676
Amino Sugar Metabolism	33	2	0.138	0.676
Gluconeogenesis	33	2	0.138	0.676
Beta-Alanine Metabolism	34	2	0.145	0.676
Aspartate Metabolism	35	2	0.152	0.676
Fatty Acid Biosynthesis	35	2	0.152	0.676
Tyrosine Metabolism	70	3	0.159	0.676
Homocysteine Degradation	9	1	0.167	0.68
Pyruvaldehyde Degradation	10	1	0.183	0.719
Taurine and Hypotaurine Metabolism	12	1	0.216	0.811
Ketone Body Metabolism	13	1	0.232	0.811
Thyroid hormone synthesis	13	1	0.232	0.811
Steroid Biosynthesis	48	2	0.248	0.838
Alpha Linolenic Acid and Linoleic Acid Metabolism	17	1	0.292	0.932
Spermidine and Spermine Biosynthesis	18	1	0.307	0.932
Nucleotide Sugars Metabolism	20	1	0.334	0.932
Catecholamine Biosynthesis	20	1	0.334	0.932
Threonine and 2-Oxobutanoate Degradation	20	1	0.334	0.932
Pantothenate and CoA Biosynthesis	21	1	0.348	0.932
Betaine Metabolism	21	1	0.348	0.932
Carnitine Synthesis	22	1	0.361	0.932
Transfer of Acetyl Groups into Mitochondria	22	1	0.361	0.932
Inositol Phosphate Metabolism	24	1	0.387	0.955
Arachidonic Acid Metabolism	67	2	0.391	0.955
Glycerolipid Metabolism	25	1	0.4	0.955
Plasmalogen Synthesis	26	1	0.412	0.961

Mitochondrial Beta-Oxidation of Long Chain Saturated Fatty Acids	28	1	0.436	0.977
Folate Metabolism	29	1	0.447	0.977
Inositol Metabolism	30	1	0.459	0.977
Lysine Degradation	30	1	0.459	0.977
Starch and Sucrose Metabolism	31	1	0.47	0.98
Citric Acid Cycle	32	1	0.481	0.982
Nicotinate and Nicotinamide Metabolism	35	1	0.512	1
Fatty Acid Elongation In Mitochondria	35	1	0.512	1
Galactose Metabolism	38	1	0.542	1
Porphyrin Metabolism	40	1	0.561	1
Propanoate Metabolism	42	1	0.579	1
Histidine Metabolism	42	1	0.579	1
Fatty acid Metabolism	43	1	0.588	1
Steroidogenesis	43	1	0.588	1
Pyruvate Metabolism	47	1	0.621	1
Valine, Leucine and Isoleucine Degradation	59	1	0.706	1
Tryptophan Metabolism	59	1	0.706	1

[a] Total Cmpd: total number of compounds in the pathway, [b] Hit: actually matched number from the data
[c] Raw p: p-value calculated from the enrichment analysis, [d] FDR: p-value adjusted using False Discovery Rate
